# Niemann-Pick Diseases: The Largest Iranian Cohort with Genetic Analysis

**Published:** 2019

**Authors:** Somayyeh Hashemian, Peyman Eshraghi, Nafi Dilaver, Hamid Galehdari, Bita Shalbafan, Rahim Vakili, Nosrat Ghaemi, Najmeh Ahangari, Jamileh Rezazadeh Varaghchi, Jawaher Zeighami, Alireza Sedaghat, Majid Aminzadeh, Mohammad Hamid, Alihossein Saberi, Fereshteh Ashtari, Ehsan Ghayoor Karimiani, Gholamreza Shariati

**Affiliations:** 1 Department of Pediatric Endocrinology and Metabolism, Akbar Hospital, Mashhad University of Medical Sciences, Mashhad, Iran; 2 Swansea University College of Medicine, Swansea University, Swansea, UK; 3 Narges Medical Genetics and Prenatal Diagnosis Laboratory, East Mihan Ave, Kianpars, Ahvaz, Iran; 4 Department of Genetics, Faculty of Science, Shahid Chamran University of Ahvaz, Ahvaz, Iran.; 5 Iran Social Security Organization, Labafinejad Hospital, Tehran, Iran; 6 Medical Genetic Research Center, Mashhad University of Medical Sciences, Mashhad, Iran; 7 Department of Molecular Genetics, Hope Generation Genetic Polyclinic, Mashhad, Iran; 8 Genetic counselling and Rehabilitation Unit, Welfare organization, South Khorasan, Iran; 9 Department of Internal Medicine, Ahvaz Jundishapur University of Medical Sciences, Ahvaz, Iran; 10 Department of Pediatrics, Ahvaz Jundishapur University of Medical Sciences, Ahvaz, Iran; 11 Department of Molecular Medicine, Biotechnology Research Center, Pasteur Institute of Iran, Tehran, Iran; 12 Department of Medical Genetics, Ahvaz Jundishapur University of Medical Sciences, Ahvaz, Iran; 13 Department of Neurology, School of Medicine, Isfahan University of Medical Science, Isfahan, Iran; 14 Razavi Cancer Research Center, Razavi Hospital, Imam Reza International University, Mashhad, Iran; 15 Division of Evolution and Genomic Sciences, Medicine and Health Sciences, University of Manchester, UK

**Keywords:** Niemann-pick disease (NPD), Genetic analysis, Autosomal recessive, Iran

## Abstract

**Objectives:**

Niemann-Pick diseases (NPD) is an autosomal recessive inherited lysosomal lipid storage disorder which occurs due to a defect in cellular cholesterol trafficking, leading to excess lipid accumulation in multiple organ systems such as the brain, lungs, spleen, and liver. SPMD1-associated disease includes classic infantile and visceral NPD type A and B respectively. Type C NPD is subacute or juvenile.

**Materials & Methods:**

During 2012-2016, the patients who had the clinical and biochemical signs and symptoms of different types of NPD, underwent genetic analysis. All patients were collected from five provinces in Iran (Razavi Khorasan, South Khorasan, Khozaestan, Isfahan and Tehran province). Sanger sequencing of the candidate genes for NPD was performed followed by bioinformatics analysis to confirm the types of NPD and to identify novel mutations. All patients underwent full clinical assessment.

**Results:**

We present two cases with NPD type A, six cases with NPD type B, and 11 cases with type C with various enzymatic defects identified in these cases. Within these 19 patients, we present 9 previously reported mutations and 10 novel mutations causing NPD.

**Conclusion:**

This study is the largest Iranian study for NPD analysis ever. Our report demonstrates that NPD has a variable age of onset and can present early in life. We investigated the clinical and genetic manifestations of a large Iranian cohort. Understanding the variable presentation of NPD will allow for clinicians to have a high index of suspicion for the disease.

## Introduction

Niemann-Pick Diseases (NPD) is a disorder that affects multiple organ systems. It has an extensive range of presenting symptoms which differ in severity. NPD is classified into four types: A, B, C1, and C2. These types are classified based on the genetic cause, clinical signs and presenting symptoms (1). NPD type A and B are also known as a SMPD1-associated disease which constitutes different clinical phenotypes of a primary sphingomyelin storage disorder resulting from acid sphingomyelinase deficiency due to SMPD1 gene mutations (2-4). The classical phenotype of NPD is often described as hepatosplenomegaly, with progressive ataxia, dystonia, and dementia. NPD type A is the most common type present in infants and is characterized by jaundice, hepatomegaly, failure to thrive, progressive deterioration of the nervous system and profound brain damage most often leading to death before 18 months of age (5). Type A is most common amongst those from Ashkenazi (eastern and central European) Jewish descent (6).

In NPD type B patients, hepatosplenomegaly is often present which may be severe in the presence or absence of signs of liver failure. Serum low-density lipoprotein (LDL)-cholesterols and triglycerides are often elevated in NPD, although high-density lipoprotein (HDL)-cholesterol is found to be at low levels. Another clinical sign present in some type B cases is a distinct cherry-red spot in the macula (7). Type B patients have no overt signs of central nervous system involvement but frequently have compromised pulmonary function (6). NPD type C can present in infancy, childhood, or adulthood. Neonates may present with severe ascites due to severe liver disease or respiratory failure as well as renal failure (8, 9). Newborns presenting without liver or pulmonary disease, often present with hypotonia and developmental delay (1).

NPD type C most commonly develops in late childhood with most patients not surviving to the second decade of life (2-4). Other phenotypes include fatal neonatal liver disease, delayed motor development and early infantile onset with hypotonia. Adult variations in the phenotype include onset of psychosis and dementia, juvenile dystonic lipidosis, DAF (downgaze paresis, ataxia, foam cells) syndrome, adult dystonic lipidosis, dystonia, and organomegaly, all of now recognized as presentations of NPD (9-11). 

In this case series, we describe 19 cases to illustrate the clinical manifestations of NPD and further discuss the variations in the genetics findings and biochemistry.

## Case Presentation

In this study, we present 19 patients with ranging types of NPD including Type A, B, C, and C1. All cases who presented, were admitted to their local hospital due to onset of health complications. Each patient underwent a complete clinical assessment (Table 1). Each case also underwent the appropriate biochemical investigations, which revealed enzymatic defects. Post-genetic counseling and molecular investigations such as Sanger sequencing were performed to confirm the clinical diagnosis of NPD and determine the type of NPD present in all patients. 

Written informed consent was obtained from all subjects and the study protocol has been approved by the Regional Ethics Committee in the field of human research, in their local universities.

Two cases had NPD type A, both of whom died in infancy. Six patients had type B NPD of which four died by 4.5 years of age and two are currently alive and under three years of age. Eleven patients had/ have NPD type C/ C1. Five of the patients were born to parents not-related and 14 to consanguineous parents (Table 1). Fourteen patients had hepatosplenomegaly and elevated liver enzymes, and 15 patients had developmental and psychomotor regression. Two cases had an auditory impairment and four with a visual impairment. In 10 cases we identified novel mutations predicted likely pathogenic.

## Discussion

In this case series, we have presented 19 different patients from different families and nationality with NPD type A, B, C, and C1. Data were consolidated for the clinical manifestations and biochemical findings as well as genetic investigations performed. This case series provides clinical data on 19 patients with NPD with 10 novel previously undescribed mutations along with formerly reported mutations. Clinical symptomatology and disease progression in NPD are markedly affected by the age of disease onset of neurological manifestations also suggested elsewhere (11).

**Table 1:** Each patients’ clinical findings including and genetic information with mutation identified

**Case Number**	**1**	**2**	**3**	**4**	**5**	**6**	**7**	**8**	**9**	**10**	**11**	**12**	**13**	**14**	**15**	**16**	**17**	**18**	**19**
**NPD Type**	A	A	B	B	B	B	B	B	C	C1	C1	C1	C1	C1	C1	C1	C1	C1	C1
**Current Age**	Died at ~6 months old	Died at 2.5 years old	Died at 3 years old	Died at 4.5 years old	Died at 3 years old	Died at 1.5 years old	Alive - 1.5 years old	Alive - 2.4 years old	Died at 2 years old	Alive - 3.5months	Alive - 6 months	Alive - 4.9 years	Died at birst	Died at 2.4 years old	Alive - 28.5 years old	Alive - 29.5 years old	Alive - 46 years old	7 months	Alive - 25 years old
**Gender**	Male	Male	Female	Male	Female	Male	Male	Female	Female	Male	Female	Male	Female	Female	Male	Female	Male	Female	Female
**Ethnicity**	Arab Iranian	Arab Iranian	Arab Iranian	Bakhtiari	Lor (Baghmalek)	Arab Iranian	Fars	Fars	Bakhtiari	Fars	Fars	Fars	Fars	Fars	Fars	Fars	Fars	Fars	Turk (Azari)
**Medical & Family History**																			
**Consanguinity of parents'**	Second cousin	First cousin	First cousin	First cousin	First cousin	First cousin	Not related	First cousin	First cousin	Not related	First cousin	First cousin	First cousin	First cousin	Not related	Not related	Not related	First cousins	Distantly related
**Other family members with NPD, if yes, Type**	N	N	N	N	N	N	N	N	N	N	N	N	Y - Type C	Y - Type C	Y - Type C	Y - Type C	Y - Type C	N	N
**Age of Diagnosis**	6 months	5 months	6 months	6 months	6 months	7 months	Unknown	Unknown	6 months	3 months	5 months	4.5 years	At birth	6 months	27 years	28 years	44 years	6 months	25 years
**Neonatal icterus (Y / N)**	N	N	Y	Y	N	Y	Y	Y	N	Y	Y	Y	N	N	N	N	N	N	N
**Failure to thrive (Y / N)**	N	N	N	N	N	N	Y	N	Y	Y	Y	Y	Y	Y	N	N	N	N	N
**Splenomegaly (Y / N)**	Y	Y	Y	Y	Y	Y	Y	Y	Y	Y	Y	Y	Y	Y	N	N	N	Y	N
**Hepatomegaly (Y / N)**	Y	Y	Y	Y	Y	Y	Y	Y	Y	Y	Y	Y	Y	Y	N	N	N	Y	N
**Any intellectual disability (Y / N)**	Y	Y	N	Y	N	N	Y	Y	N	Y	Y	Y	NA	Y	Y	Y	Y	N	Y
**Developmental regression (Y / N)**	Y	Y	Y	Y	Y	Y	Y	Y	Y	Y	Y	Y	NA	Y	N	N	Y	Y	Y
**Gaze paresis (Y / N)**	N	N	N	N	N	N	N	N	N	N	N	N	NA	Y	Y	Y	Y	N	Y
**Epilepsy (Y / N)**	N	Y	N	N	N	N	N	N	N	N	N	N	NA	N	N	N	Y	N	Y
**Mental illness (Y / N)**	Y	Y	Y	N	N	N	N	N	N	N	N	N	NA	Y	Y	Y	Y	Y	Y
**Cardiac abnormalities (Y / N)**	N	N	N	N	N	N	N	N	N	RVH	N	N	NA	N	N	N	N	N	N
**Hearing impairment (Y / N)**	N	N	N	N	N	N	N	N	N	N	N	N	NA	Y	N	N	N	N	N
**Visual impairment, if yes, specify**	N	Cherry-red spot	N	N	N	N	Cherry-red spot	N	Cherry-red spot	N	N	N	NA	Y	N	N	N	N	N
**Genetics**																			
**Gene**	SMPD1	SMPD1	SMPD1	SMPD1	SMPD1	SMPD1	SMPD1	SMPD1	NPC1	NPC1	NPC1	NPC1	NPC1	NPC1	NPC1	NPC1	NPC1	NPC1	NPC1
**Mutation**	c.740delG	c.740delG	c.108delG	c.98_144delTGGGCCTGGTGCTGGCGCTGGCGCTGGCGCTGGCGCTGGCGCTGGCT	c.1110delT	c.573delT	c.1390G>T	c.1524G>A	c.2920_2923delCCTG	c.2740 T>A	c.1415T>C	c.1415T>C	c.3478-6T>A	c.3478-6T>A	c.2476_2484del	c.2476_2484del	c.2476_2484del	c.960_961dup	c.409A>G
**Amino acid Substitution**	G247Afs*10	G247Afs*10	L37Wfs*40	M33Tfs*3	L371Ffs*14	S192Afs*65	p.Glu464Ter	P.Gly508Gly -no AA change	C976Ffs*6	p.Cys 914 Ser	p.Leu472Pro	p.Leu472Pro	Unknown	Unknown	p.Ser826_Leu	p.Ser826_Leu	p.Ser826_Leu	p.(Ala321Glyfs*16)	Thr137Ala
**Novel mutation (Y / N)**	N	N	Y	Y	Y	N	Y	N	Y	Y	Y	Y	N	N	N	N	N	Y	Y
**Pathogenic (Y / N)**	Y	Y	Likely	Likely	Likely	Y	Likely	Y	Likely	Likely	Likely	Likely	Y	Y	N	Y	Y	Y	Y

Overall, the main complication present in most cases was liver disease, affecting 14 patients out of 19. Liver disease is known to be a cause of significant morbidity and mortality in NPD. The diagnosis of NPD type C should be considered in patients with unexplained neonatal hepatitis especially in the presence of splenomegaly (12). NPD should also be high on the differential diagnosis in the presence of systemic symptoms such as neonatal jaundice and isolated splenomegaly, neurological symptoms such as dystonia, dementia, cataplexy and supra nuclear gaze palsy which may occur in patients (13). In this case series, we have presented 10 patients who were female and 9 males. All cases with type A or B NPD had mutations in *SMPD1* and all those with NPD type C on *NPC1*. Identification of mutations in *NPC1* is challenging due to the relatively large nature of the gene and majority of the mutations being private (12). 

There are 3 types of NPD that the primary biological defect is different (13). NPD types A and B are autosomal recessive lysosomal storage diseases caused by the deficient activity of acid sphingomyelinase due to mutations in the *SMPD1*. Genetic variants which are considered as disease causing are distributed in *SMPD1* gene. Most of these variants are missense or frameshift mutations (14). In this case series, eight of the cases had mutations in *SMPD1* predicted to be pathogenic or likely pathogenic. Studies have shown that the *SMPD1* is preferentially expressed from the maternal chromosome (15). In Iran, the first molecular diagnosis of NPD type A was reported. It had detected a novel deletion in SMPD1 gene (16). A novel mutation in exon 9 of NPC1 gene was reported from Khorasan Province, Iran (17). 

Consanguineous marriage is common upon our region (18) and consanguinity was observed between the parents of 14 cases; however, both developmental and psychomotor regression were observed in all but two cases presented. Mongolian spots and cherry-red spot were present in three cases. Case 13 and 14 were deceased prior to the final genetic investigations were performed. 

**In conclusion, **these findings in NPD have clinical implications for genetic counseling. Our study provides a large number of patients with varying presentations and novel mutations. In suspected NPD, clinicians should confirm the carrier status of both parents and evaluate other first-degree relatives to provide families with accurate genetic counseling.
